# Control of Microbial Opsin Expression in Stem Cell Derived Cones for Improved Outcomes in Cell Therapy

**DOI:** 10.3389/fncel.2021.648210

**Published:** 2021-03-18

**Authors:** Marcela Garita-Hernandez, Antoine Chaffiol, Laure Guibbal, Fiona Routet, Hanen Khabou, Luisa Riancho, Lyes Toualbi, Serge Picaud, José-Alain Sahel, Olivier Goureau, Jens Duebel, Deniz Dalkara

**Affiliations:** ^1^Institut de la Vision, Sorbonne Université, Paris, France; ^2^CHNO des Quinze−Vingts, DHU Sight Restore, Paris, France; ^3^Department of Ophthalmology, The University of Pittsburgh School of Medicine, Pittsburgh, PA, United States; ^4^Department of Ophthalmology, University Medical Center Göttingen, Göttingen, Germany

**Keywords:** human induced pluripotent stem cell, human retinal organoid, cones, optogenetics, vision restoration, cell therapy, promoter

## Abstract

Human-induced pluripotent stem cell (hiPSC) derived organoids have become increasingly used systems allowing 3D-modeling of human organ development, and disease. They are also a reliable source of cells for transplantation in cell therapy and an excellent model to validate gene therapies. To make full use of these systems, a toolkit of genetic modification techniques is necessary to control their activity in line with the downstream application. We have previously described adeno-associated viruse (AAV) vectors for efficient targeting of cells within human retinal organoids. Here, we describe biological restriction and enhanced gene expression in cone cells of such organoids thanks to the use of a 1.7-kb L-opsin promoter. We illustrate the usefulness of implementing such a promoter to enhance the expression of the red-shifted opsin Jaws in fusion with a fluorescent reporter gene, enabling cell sorting to enrich the desired cell population. Increased Jaws expression after transplantation improved light responses promising better therapeutic outcomes in a cell therapy setting. Our results point to the importance of promoter activity in restricting, improving, and controlling the kinetics of transgene expression during the maturation of hiPSC retinal derivatives. Differentiation requires mechanisms to initiate specific transcriptional changes and to reinforce those changes when mature cell states are reached. By employing a cell-type-specific promoter we put transgene expression under the new transcriptional program of mature cells.

## Introduction

The generation of human retinal organoids has opened up new ways to study the brain and retinal development and evolution, as well as to model and treat neurodegenerative disorders (Ahmad et al., 2019; Gagliardi et al., 2019). Retinal organoids are multicellular 3D structures that mimic certain aspects of the cytoarchitecture and cell-type composition of the human retina. These structures are generated by the differentiation of Human-induced pluripotent stem cells (hiPSCs) or embryonic stem cells (ESCs; Llonch et al., 2018; Ahmad et al., 2019; Lidgerwood et al., 2019). However, even the best accomplished 3D model of retinal development carries some limitations and can benefit from genetic modifications to control cellular functions. The ability to genetically modify retinal organoids is essential for their utility as disease models as well as their therapeutic use for regenerative medicine. Genetic modification is a powerful tool that allows for the introduction of alterations ranging from small changes in the genome to the removal or integration of entire genes to the expression of exogenous genes to control cellular function (Garita-Hernandez et al., 2016). This enables researchers to investigate individual genes relating to cellular functions, and interactions. Moreover, drugs (small molecule or gene-based) can be tested in a larger variety of disease states and in different genetic environments (Kalatzis et al., 2013; Cereso et al., 2014; Garita-Hernandez et al., 2018; Quinn et al., 2018). Any method of genetic modification of hiPSC derivatives needs to address three key issues: (i) the ultimate purpose of the modification (investigative study or therapy); (ii) the stage of differentiation and the bioavailability of receptors; and (iii) the proportion of cells target of the genetic transformation. For non-integrative genetic modifications to be stable, it should be made once the cell cycle arrest has been induced, circumventing cell divisions. The delivered genetic cargo is then maintained even in the absence of chromosomal integration and the transgene expression is dependent on the recruitment of transcription factors by the promoter sequence preceding the transgene.

Due to the cellular heterogeneity of retinal organoids, the third issue concerns the cell type(s) within the organoid that is/are subject to genetic modifications. Approaches can either target cells indiscriminately, regardless of their location and cell type (Garita-Hernandez et al., 2018, 2020; Gonzalez-Cordero et al., 2018) or, contrariwise, target a subset of cells. The latter can be achieved by biologically restricting the genetic modification to specific cell types by the use of cell type-specific promoters to drive transgene expression (Macé et al., 2015; El-Shamayleh et al., 2016; Chaffiol et al., 2017; Juettner et al., 2018; Khabou et al., 2018). In this work, we targeted to achieve high-level microbial opsin expression in cones derived from human retinal organoids to restore light responses in blind mice following transplantation of these cells. For this type of genetic modification, one of the most important aspects to consider is the mode of gene delivery and control of transgene expression. The two most widely used methods in the case of organoids are viral delivery *via* adeno-associated viruses (AAVs) and non-viral methods such as electroporation (Fischer et al., 2019). AAVs have been successfully used in transducing retinal organoids by simple addition to the cell culture medium, which results in gene expression throughout the entire organoid (Garita-Hernandez et al., 2018, 2019, 2020; Gonzalez-Cordero et al., 2018; Quinn et al., 2018). This is a reliable approach to broadly target the whole organoid, but it must be refined to meet the needs of the downstream application.

The spectrum of potential applications has so far ranged from the simple expression of fluorescent marker proteins (Gagliardi et al., 2018) to the modeling of disease conditions (Artegiani et al., 2020). Yet, significant untapped potential remains for the future use of gene delivery to retinal organoids in disease modeling and therapy (Dalkara et al., 2016). Over the last 5 years, efforts have been directed to the transplantation of photoreceptors derived from 3D retinal organoids (Gonzalez-Cordero et al., 2017; Gagliardi et al., 2018; Lakowski et al., 2018; Collin et al., 2019; Aboualizadeh et al., 2020) resulting in different levels of success but the formation of light-sensitive outer segments (Mandai et al., 2017; Iraha et al., 2018) and interaction with retinal pigment epithelium (RPE), which is necessary for the appropriate functioning of photoreceptor cells (Strauss, 2005) remain challenges for cell replacement with therapeutic outcomes. Microbial opsins can circumvent these issues as we have recently proposed (Garita-Hernandez et al., 2019). Using the hyperpolarizing microbial opsin Jaws, we have previously conferred light sensitivity to hiPSC-derived cones. After transplantation of optogenetically-transformed cones, we observed restoration of light responses in blind mice both at the retinal and behavioral levels under very bright light (Garita-Hernandez et al., 2019). Here, we demonstrate that the success of this approach can be increased by expressing the microbial opsin selectively and at a high level in the desired cell population within the organoid. The use of a strong and cell-type-specific promoter allows to isolate and enrich such population *via* fluorescence-activated cell sorting. We hypothesize that an increase in transgene expression occurs *via* increased availability of cone-specific transcription factors as cells mature in the subretinal space. Enhanced microbial opsin expression contributes to better light sensitivity and temporal resolution of light responses in the transplanted cones paving the way to better therapeutic efficacy in vision restoration. To our knowledge this is the first time a molecular strategy has been used to overcome issues related to the isolation of a target hiPSC-derived cell population and control of transgene expression within these cells, thereby improving the response amplitude and the kinetic profile of light responses *via* a microbial opsin.

## Materials and Methods

### Maintenance of hiPSC Culture

All experiments were performed using hiPSC-2 and hiPSC-5f cell lines, previously established from human fibroblasts and Müller glial cells respectively (Reichman et al., 2014; Slembrouck-Brec et al., 2019). Cells were kept at 37°C, under 5% CO_2_/95% air atmosphere, 20% oxygen tension, and 80%–85% humidity. Colonies were cultured in feeder-free conditions as previously described (Reichman et al., 2017) with Essential 8^TM^medium (Thermo Fisher Scientific) in culture dishes coated with truncated recombinant human Vitronectin (Thermo Fisher Scientific). The medium was changed every day and the cells were passaged once a week when reaching 70% of confluency.

### Differentiation of Human iPSCs Into Retinal Organoids

Optimization of previous protocols (Reichman et al., 2017) allowed the generation of retinal organoids from hiPSC. In brief, hiPSC cell lines were expanded until 80% confluence in Essential 8^TM^ medium before switched in Essential 6^TM^ medium (Thermo Fischer Scientific). After 3 days, cells were cultured in a *Proneural medium*. On day 28, NR-like structures grew out of the cultures and were mechanically isolated and further cultured in a 3D system in *Maturation medium* until day 70 of differentiation. Table 2 summarizes media formulation. Floating organoids were passed to 6 well-plates (10 organoids per well) and supplemented with 10 ng/ml FGF2 (Preprotech) until day 35. Additionally, between day 42 and day 49, 10 μM DAPT (Selleckchem) was added to the *Maturation medium* to promote the photoreceptor commitment of retinal progenitors. The medium was changed every 2–3 days (Figure 2; Garita-Hernandez et al., 2018).

**Table 1:** Animal details used to generate figures.

Figure	Strain	Experimental group	Experiment	Transplant age (weeks)	Analysis age (weeks)	Number of mice *(N)*
2-C/D	rd1−/−	Enriched human Jaws-cones	Fundus	7	9	23
2-E	rd1−/−	Enriched human Jaws-cones	Fundus	7	10	23
2-F	rd1−/−	Enriched human Jaws-cones	2-photon	7	11	1
2-G	rd1−/−	Non transplanted	IHC	-	7	1
2-H/I	rd1−/−	Enriched human Jaws-cones	IHC	7	12	5
3	rd1−/−	Enriched human Jaws-cones	Patch clamp	7	12	5
4	rd1−/−	Enriched human Jaws-cones	Patch clamp	7	12	2
Supplementary Figure 1A	rd1−/−	Enriched human Jaws-cones	IHC	7	12	5
Supplementary Figure 1B	rd1−/−	Enriched human Jaws-cones	IHC	7	12	5
Supplementary Figure 1C	rd1−/−	Enriched human Jaws-cones	IHC	7	12	5

**Table 2:** Media formulation for 100 ml total volume.

Medium	Formulation (100 ml)
Maintenance of hiPSCs	Essential 8^TM^ Basal medium (Thermo Fischer Scientific)– 98 ml. Essential 8^TM^ Supplement 50X (Thermo Fischer Scientific)–2 ml.
Proneural medium	Essential 6^TM^ medium (Thermo Fischer Scientific)–95ml. Penicillin/Streptomycin (Thermo Fischer Scientific)–100 μl. N2 supplement (Thermo Fischer Scientific)–5 ml.
Maturation medium	DMEM-F12^ TM^ (Thermo Fischer Scientific)–97 ml. B27 Supplement (Thermo Fischer Scientific)–2 ml. MEM Non-Essential Amino Acids Solution 100X (Thermo Fischer Scientific)–1 ml. Penicillin/Streptomycin (Thermo Fischer Scientific)–100 μl.
Ringer Solution	NaCl 155 mM, KCl 5 mM, CaCl2 2 mM, NaCl2 1 mM, NaH2PO4 2 mM, HEPES 10 mM, glucose 10 mM.

**Figure 1:**
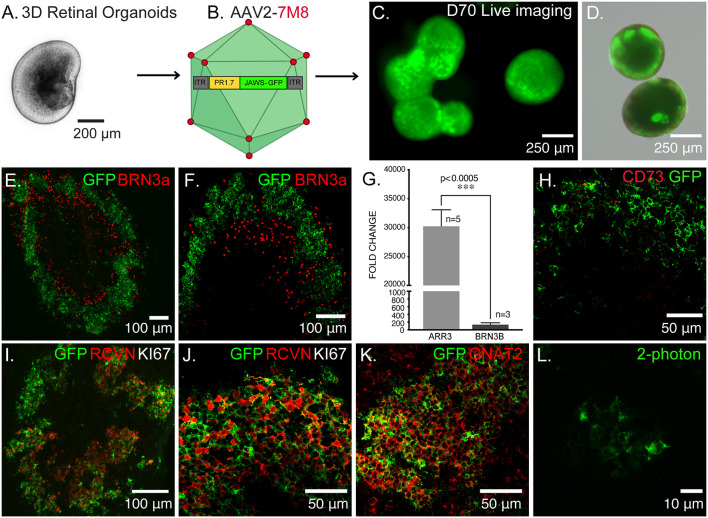
Optogenetic engineering of retinal structures derived from human induced pluripotent stem cells (hiPSCs). **(A)** Bright-field/epifluorescence image of a hiPSC-derived retinal organoid after mechanical isolation on day 30 of differentiation. **(B)** Schematics of the adeno-associated viruses (AAV)-mediated strategy used to engineer 3D retinal organoids, AAV2–7m8-PR1.7-Jaws-GFP was used to transduce cone photoreceptors on day 44 of differentiation. **(C)** Live GFP fluorescence observed after prolong culture of retinal organoids showing strong and long-term expression of the transgene. **(D)** Merge of bright-field/epifluorescence and live GFP fluorescence images observed in 70-days old organoids. **(E)** Immunofluorescence (IF) analysis of a representative retinal organoid at day 70 of differentiation, depicting a thick layer of photoreceptors immunoreactive for GFP and some sparse ganglion precursors cells identified by BRN3a. **(F)** Magnification of the IF analysis in **(D)** in another organoid. **(G)** Real-time qRT-PCR analysis of cone specific marker, Cone arrestin (ARR3) and retinal ganglion cell specific marker BRN3B. *N* = number of biological replicates. Values are mean ± SEM. Error bars are SEM. Statistical significance assessed using Mann–Whitney Student’s test (***p < 0.0005). **(H)** IF analysis of optogenetically engineered retinal organoids at day 70 showing Jaws+ cells do not express CD73 photoreceptor surface marker. **(I)** IF analysis confirming postmitotic photoreceptor identity of GFP positive cells, showing they are immunoreactive for the photoreceptor-specific marker Recoverin (RCVN) and complete absence of proliferation marker KI67. **(J)** Magnification of the IF analysis in **(I)** in another organoid. **(K)** IF analysis of confirming the cone identity of GFP positive cells, showing colocalization with cone-specific marker GNAT2. **(L)** 2-Photon laser image of GFP+ cells inside a retinal organoid at D70 of differentiation. Images correspond to representative retinal organoids from at least four different biological replicates, scale bar: **(C,D)** = 250 μm, **(E,F,I)** = 100 μm, **(H,J,K)** = 50 μm, **(L)** = 10 μm.

**Figure 2:**
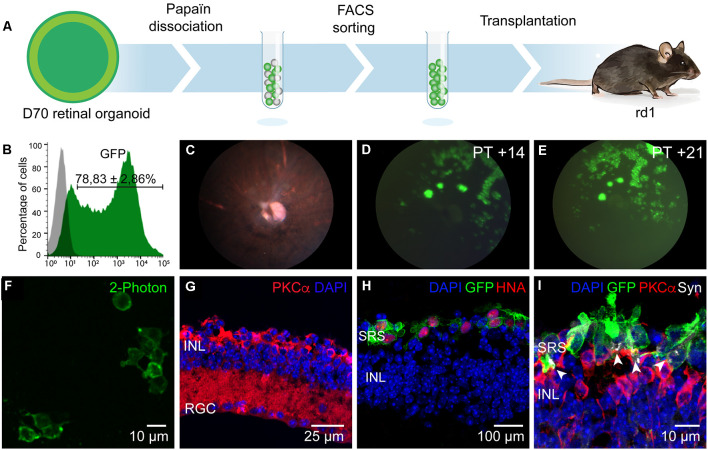
Enrichment and transplantation of optogenetically-engineered cones. **(A)** Schematics of the general transplantation strategy. Human iPSC-derived retinal organoids were Papain-dissociated and Jaws-enriched expressing cells were transplanted subretinally into blind rd1 mice. **(B)** Representative histogram of flow cytometry analysis performed to determine Jaws-expressing cells within the retinal organoids (*N* = 8 biological replicates, *n* = 10,000 cells gated). **(C)** Fundus imaging analysis of representative rd1 mice retinae after 14 days of transplantation. **(D,E)** Representative fluorescent fundus images of rd1 mice in panel **(C)** showing Jaws-expressing cells 14 **(D)** and 21 **(E)** days after subretinal transplantation. **(F)** 2-Photon laser image of GFP+ cells in a rd1 transplanted retina. **(G)** Histological analysis of 7-week-old mice retina showing complete absence of photoreceptors layers, corresponding to the time of transplantation. Remaining cells are immunoreactive for bipolar specific marker PKCα. **(H,I)** IF analysis of vertical cryosections of rd1 mice retinae after transplantation of Jaws-enriched expressing cells (GFP). **(H)** Transplanted Jaws-cones depicted as GFP+ cells in rd1 mice were also immunoreactive for human marker HNA (red). **(I)** Transplanted cones overlie host PKCα bipolar cells and expressed synaptic marker Synaptophysin (Syn, white arrows) All images are representative of at least five different transplants. Animal details for each transplantation are provided in Table 1. Scale bars: **(F** and **I)** = 10 μm, **(G)** = 25 μm, **(H)** = 100 μm. INL, inner nuclear layer, RGC, retinal ganglion cells; SRS, subretinal space.

### AAV Vector Production

Recombinant AAV2–7m8 were produced as previously described using the co-transfection method and purified by iodixanol gradient ultracentrifugation (Choi et al., 2007). Concentration and buffer exchange was performed against PBS containing 0.001% Pluronic. AAV vector stocks titers were then determined by the real-time quantitative PCR titration method (Aurnhammer et al., 2012) using SYBR Green (Thermo Fischer Scientific).

### Infection of Retinal Organoids With AAV Encoding Jaws-GFP

Retinal organoids were transduced at day 44 using the recombinant AAV2–7m8 capsid variant (Dalkara et al., 2013) carrying the hyperpolarizing chloride pump Jaws (Chuong et al., 2014) fused to the fluorescent reporter GFP under the control of the cone-specific promoter PR1.7 (Ye et al., 2016). One single infection was performed by direct addition in *Proneural medium* (Table 2) of 5 × 10^10^ vg per organoid in 6-well plates containing 10–12 organoids. The medium was changed 72 h after infection.

### Single-Cell Dissociation of Retinal Organoids

Retinal organoid dissociation was done with two units of pre-activated papain at 28.7 u/mg (Worthington) in Ringer solution. The samples were incubated 25 min at 37°C with several pipetting steps to obtain a homogenous suspension. The cell solution was filtered with a 40 μm filter (Miltenyi Biotec, Germany) and centrifuged for 5 min at 800 rpm. The pellet was resuspended at the desired concentration in a *Proneural medium* (Table 2) for cell transplantation.

### Flow Cytometry Analysis

Dissociated retinal organoids were filtered with a 30 μm filter (Miltenyi, Bergisch Gladbach, Germany) and fixed for 10 min at 4°C with 4% paraformaldehyde. Cells were washed in PBS. The samples were analyzed by flow cytometry and at least 10,000 events were examined in each experiment using the FACSCalibur system (BD Biosciences, Allschwil, Switzerland). The number of GFP-positive cells within the gated population was measured using CellQuest^TM^ Pro (BD Biosciences) software. Non-infected organoids serve as controls.

### Fluorescence-Activated Cell Sorting (FACS)

Sorting of GFP positive cells was performed from Papain single-cell dissociated retinal organoids. Dissociated cells were passed through a 30 μm filter (Miltenyi, Bergisch Gladbach, Germany) and resuspended in PBS/0.5% FBS/5 mM EDTA (2.10^6^ cells/ml). The GFP positive cells were sorted with a MoFlo Astrios EQ FACS system (Beckman Coulter, Villepinte, France) in an enrichment mode.

### Animals

Retinal degeneration 1 (rd1) mice line (C3H *rd/rd*) was kindly provided by Dr. Thierry Leveillard and used as a cell recipient. Rd1 mice photoreceptor cells degenerate to a single row of cones by P20 (Farber and Lolley, 1974; Lavail and Sidman, 1974; Bowes et al., 1990). All mice were housed under a 12-h light-dark cycle with free access to food and water (certified animal facility of the “Institut de la Vision”; agreement number A751202). All experiments were carried out in strict accordance with the Association for Research in Vision and Ophthalmology statement for animal research in ophthalmology. Moreover, all procedures were approved by the local animal experimentation ethics committee (Charles Darwin Ethical Committee for Animal Experimentation C2EA-05) in strict accordance with French and European regulations for animal use in research (Directive 2010/63/EU).

### Transplantation Procedures

Mice were anesthetized by intraperitoneal injection of ketamine (50 mg/kg) and xylazine (10 mg/kg) and placed on a heating pad to maintain body temperature at 37°C. Pupils were dilated with 0.5% mydriaticum (Thea) and a blunt 34-gauge needle was inserted tangentially through the conjunctiva and sclera. Using a Hamilton syringe, 1 μl total volume of cell suspension containing approximately 100,000 (97,667 ± 4,041) GFP+ cells were delivered into the subretinal space of each eye. A drop of antibiotic, Ophtalon (Tvm) was applied and mice were placed into a warm chamber after the surgery until their awakening. After transplantation, the mice were under oral treatment of 210 mg/L Cyclosporine A (Atopica^TM^, Novartis) administrated in the drinking water. To improve stability Cyclosporine A was prepared in 5% glycosylated water and changed three times per week.

### Fundus Analysis

Transplanted eyes were examined with a fluorescent fundus camera (MicronII, Phoenix Laboratories) weekly. Mice were sedated with 5% isofluorane and kept in 2% isofluorane during the experiment. The pupils were dilated with a drop of 0.5% mydriaticum (Thea) and 5% neosynephrine (Faure). The eyes were kept hydrated with a drop of Lubrithal (Dechra).

### Cryosectionning and Immunohistochemistry

Ten micrometer-thick organoid sections were obtained with a Cryostat Microm and mounted on Super Frost Ultra Plu^®^ slides (Menzel Gläser). Samples were washed in PBS to remove the rest of O.C.T. and then permeabilized in PBS containing 0.5% Triton^®^ X-100 for 1 h at RT. Blocking was done with PBS containing 0.2% gelatin and 0.25% Triton X-100 for 30 min at RT. Incubation with primary antibodies (Table 3) was performed overnight at 4°C. Several washes with PBS containing 0.25% Tween20 were performed before incubation with Fluorochrome-conjugated secondary antibodies (Table 4) and DAPI (4’-6’-diamino-2-phenylindole, dilactate; Invitrogen-Molecular Probe; 1/1,000 dilution) to counterstain the nuclei for 1 h at RT. Samples were further washed in PBS and dehydrated with 100% ethanol before mounting using fluoromount Vectashield (Vector Laboratories).

**Table 3:** Primary antibodies.

Antibody	Reference	Specie/Clonality	Dilution
BRN3a	Millipore MAB1585	Mouse Monoclonal	1/250
CD73	Biolegend 344002	Mouse Monoclonal	1/100
GFP	Abcam ab13970	Chicken Polyclonal	1/500
GNAT2	Santa Cruz sc-390	Rabbit Polyclonal	1/200
hCAR	Gift from Cheryl Craft	Rabbit Polyclonal	1/20,000
HNA	Millipore MAB4383	Mouse Monoclonal	1/200
KI67	BD Pharmagen BD4127608	Mouse Monoclonal	1/200
PKCα	Santa Cruz sc-208	Rabbit Polyclonal	1/100
Recoverin	Millipore AB5585	Rabbit Polyclonal	1/2,000
Ribeye (Anti-CtBP2)	BD Biosciences 612044	Mouse Monoclonal	1/500
Synaptophysin	Sigma S5768	Mouse Monoclonal	1/200
vGlut1	Abcam ab77822	Rabbit Polyclonal	1/500

**Table 4:** Secondary antibodies.

Antibody	Reference	Dilution
Alexa Fluor-488 Donkey anti mouse	Thermo Fischer Scientific (A21202)	1/500
Alexa Fluor-488 Donkey anti rabbit	Thermo Fischer Scientific (A21206)	1/500
Alexa Fluor-488 Goat anti chicken	Thermo Fischer Scientific (A11039)	1/500
Alexa Fluor-546 Donkey anti mouse	Thermo Fischer Scientific (A10036)	1/500
Alexa Fluor-546 Donkey anti rabbit	Thermo Fischer Scientific (A10040)	1/500
Alexa Fluor-647 Donkey anti mouse	Thermo Fischer Scientific (A31571)	1/500
Alexa Fluor-647 Donkey anti rabbit	Thermo Fischer Scientific (A31573)	1/500

### Image Acquisition

Immunofluorescence was observed using a Leica DM6000 microscope (Leica microsystems, Wetzlar, Germany) equipped with a CCD CoolSNAP-HQ camera (Roper Scientific, Vianen, Netherlands) or using an Olympus FV1000 (inverted IX2 and upright BX2) laser scanning confocal microscopes equipped with a GaAsP PMT detector with 405, 488, 515, and 635 nm pulsing lasers. The images were acquired sequentially with the step size optimized based on the Nyquist–Shannon theorem. The analysis was conducted in FIJI (NIH). Images were put into a stack, Z-sections were projected on a 2D plane using the MAX intensity setting in the software’s Z-project feature, and the individual channels were merged.

### Two-Photon Imaging and Electrophysiological Recordings

A custom-made two-photon microscope equipped with a 25× water immersion objective (XLPlanN-25 × -W-MP/NA1.05, Olympus) and a pulsed femtosecond laser (InSight^TM^ DeepSee^TM^—Newport Corporation) were used for imaging and targeting AAV-transduced fluorescent cells (Jaws-GFP+ or GFP+ cells) in retinal organoids or in C3H *rd/rd* (rd1) mice retinae. Two-photon images were acquired using the excitation laser at a wavelength of 930 nm. A CCD camera (Hamamatsu Corp.) was used to visualize cells under infrared light, and images were processed offline using ImageJ (NIH). Retinal organoids were placed in the recording chamber of the microscope at 36°C in oxygenated (95% O_2_/5% CO_2_) Ames medium (Sigma–Aldrich) during the whole experiment. Ames’ Medium is a powder mixture of 42 different components, in which sodium bicarbonate is later added, its composition was initially formulated to support retinal tissue in short-term cultures (Ames and Nesbett, 1981). Transplanted mice were sacrificed by CO_2_ inhalation followed by quick cervical dislocation, and eyeballs were removed. Retinae from rd1 mice were isolated in oxygenated (95% O_2_/5% CO_2_) Ames medium and whole-mount retinas with ganglion cell side down were placed in the recording chamber of the microscope at 36°C for the duration of the experiment for live two-photon imaging and electrophysiology.

For patch-clamp recordings, AAV-transduced fluorescent cells were targeted with a patch electrode under visual guidance using the reporter tag’s fluorescence. Whole-cell recordings were obtained using the Axon Multiclamp 700B amplifier (Molecular Device Cellular Neurosciences). Patch electrodes were made from borosilicate glass (BF100-50-10, Sutter Instrument) pulled to 7–8 MΩ and filled with 115 mM K+ Gluconate, 10 mM KCl, 1 mM MgCl_2_, 0.5 mM CaCl_2_, 1.5 mM EGTA, 10 mM HEPES, and 4 mM ATP-Na2 (pH 7.2). Photocurrents were recorded while voltage-clamping cells at a potential of −40 mV. Cells were also recorded in the current-clamp configuration to monitor the membrane potential during light stimulations and measure the resting membrane potential.

### Light Stimulation of Jaws-Positive and Control Cells

Light-triggered responses from Jaws-GFP+ cells were measured in donor cells in retinal organoids before transplantation and *ex-vivo* in transplanted mice retinae, control cells expressing only GFP were also recorded in both conditions (in organoids and after transplantation). To measure light responses (photocurrents or changes in cells membrane potential) a monochromatic light source was used (Polychrome V, TILL photonics). Photocurrent and voltage hyperpolarization of cells in organoids and rd1 transplanted retinae were recorded as a function of light intensities ranging between 10^14^ and 3.1 × 10^17^ photons.cm^−2^.s^−1^ at 590 nm. To measure the activity spectrum of Jaws, 300 ms light flashes ranging from 400–650 nm (25 nm steps; interstimulus interval 1.5 s) were used at a constant light intensity of 8 × 10^16^ photons.cm^−2^.s^−1^. This light source was used at a constant wavelength of 590 nm to generate light pulses at different frequencies (ranging from 2 to 25 Hz) and durations (from 20 ms to 4 s) to determine the temporal response properties of transplanted cells expressing Jaws. Stimulation and analysis were performed using custom-written software in Matlab (Mathworks) and Labview (National Instruments) and output light intensities were calibrated using a spectrophotometer (USB2000+, Ocean Optics). Both retinal organoids and retinae were dark-adapted for at least 45 min before electrophysiological recordings.

## Results

### Generation of Optogenetically-Transformed Cone Photoreceptors

Using our previously published protocol of differentiation (Garita-Hernandez et al., 2018, 2019) we have generated cone-enriched retinal organoids in only 70 days. We have followed the 2D/3D protocol of Reichman et al. (2017) until mechanical isolation of retinal organoids (Figure 1A). 3D retinal organoids were allowed to grow in the presence of basic fibroblast growth factor-2 (FGF2) for a week and then cell cycle arrest was induced. This was performed by adding the gamma-secretase inhibitor DAPT, for a week from day 42 of differentiation. To favor differentiation towards cone lineage DAPT was added at the time point corresponding to the onset of cone arrestin (CAR) positive cells within the organoids (Garita-Hernandez et al., 2019). 3D retinal organoids were optogenetically-transformed with the recombinant AAV2-7m8 capsid variant selected for its improved performance in retinal organoids when compared to other capsids also known for their tropism towards cones (Garita-Hernandez et al., 2020). AAV2-7m8 vector carried the hyperpolarizing chloride pump Jaws (Chuong et al., 2014), under the control of the 1.7-kb cone-specific L-opsin promoter PR1.7 (Ye et al., 2016; Figure 1B). This vector-promoter combination has proven to be very efficient for gene delivery in non-human primate and human cone cells, including those in 3D retinal organoids (Khabou et al., 2018). Jaws was chosen over other hyperpolarizing microbial opsins, based on its enhanced expression levels and improved membrane trafficking in human cells (Chuong et al., 2014; Garita-Hernandez et al., 2018). The retinal organoids were transduced with AAV on day 44 of differentiation corresponding to the onset of cone-specific gene expression (Garita-Hernandez et al., 2019). We observed a strong and long-lasting expression of Jaws-GFP in retinal organoids, up to 30 days after infection (Figures 1C,D). GFP positive cells were organized in a layer in the outer part of the day 70 retinal organoids (Figures 1E,F) as a distinct major population identified as cones by the colocalization with the photoreceptor marker Recoverin (Figures 1I,J) and the cone-specific marker GNAT2 (Figure 1K). Additionally, no Jaws-GFP cells colocalize with the ganglion cell marker Brn3A (Figures 1E,F). Gene expression analysis by quantitative RT-PCR also confirmed a greater expression of cone-specific ARRESTIN in comparison with the ganglion cell marker BRN3b in our organoids (Figure 1G). Immunofluorescence analysis showed Jaws-GFP+ cells do not express the photoreceptor cell surface marker CD73 at day 70 of differentiation (Figure 1H) and no expression of the proliferation marker, Ki67 was found in day 70 organoids, excluding the presence of remaining retinal progenitor cells or tumorigenic pluripotent cells within the organoids (Figures 1I,J). These results show that using an efficient capsid promoter combination such as AAV2-7m8-PR1.7, is possible to drive a high-level specific expression of Jaws opsin in cones derived from retinal organoids.

### Selection and Transplantation of Jaws-Expressing Cone Photoreceptors

Jaws-GFP engineered 3D organoids were dissociated with papain and Jaws expressing cells were selected by FACS using the endogenous GFP expression. Sorted cells were then transplanted *via* subretinal injections into blind rd1 mouse retinas lacking the outer nuclear layer (7 weeks old rd1 mice; Figure 2A). A fraction of 78.83 ± 2.86% of cells were GFP+ (*n* = 20 organoids, *N* = 3 differentiations; Figure 2B) and approximately 100,000 cells were injected in each eye of rd1 mice in a 1 μl volume. Using *in vivo* eye fundus imaging, we detected the widespread presence of Jaws-GFP+ cells as early as 7 days after injection (Figure 2D) and transgene expression levels increased in the transplanted cells until 21 days post-transplantation (Figure 2E). At the time of transplantation, there is no detectable outer nuclear layer in the rd1 retinae (Figure 2G). Three weeks after transplantation injected eyes were dissected and flat-mount retinas were observed live under our two-photon microscope. We found abundant Jaws-GFP positive cells within the organoids, before transplantation (Figure 1L) and in transplanted retinas (Figure 2F). We fixed and cryosectioned these retinas to perform histological analysis of the transplanted region. Cross-sections of host retinas showed that all GFP positive cells corresponded to human cone cells as evidenced by colocalization with the human nuclear marker HNA (Figure 2H) and the cone-specific marker cone ARRESTIN, hCAR (Supplementary Figure 1). Immunofluorescence against the bipolar cell marker, PKCα, demonstrated close contact between the transplanted Jaws-GFP+ cones and the underlying host inner retina as well as the capability to form Synaptophysin-mediated synapses (Figure 2I). Jaws-GFP+ cells also expressed vesicular glutamate transporter 1 (vGluT1), which packs glutamate into synaptic vesicles, at the synapses. Immunohistochemistry against RIBEYE confirmed the presence of ribbon synapses (Supplementary Figure 1). Morphological analysis of the transplanted cells revealed Jaws-expressing cells lack of outer segment as previously reported (Garita-Hernandez et al., 2019). Our strategy is an alternative approach to the CD73 MACS separation (Gagliardi et al., 2018), which cannot be performed in organoids younger than 100 days old due to lack of expression of the cell surface marker, CD73. This was confirmed at the time of FACS sorting when immunofluorescence analysis showed Jaws-GFP+ cells do not express CD73 (Figure 1H). Our results show that Jaws-GFP expression driven by PR1.7 promoter can be used to successfully isolate a transplantable population of cones from day 70 organoids, which integrate with the host retina forming synaptic connections with the host bipolar cells.

### Transplanted Jaws-Expressing Cones Respond to Light Stimulus

**Figure 3:**
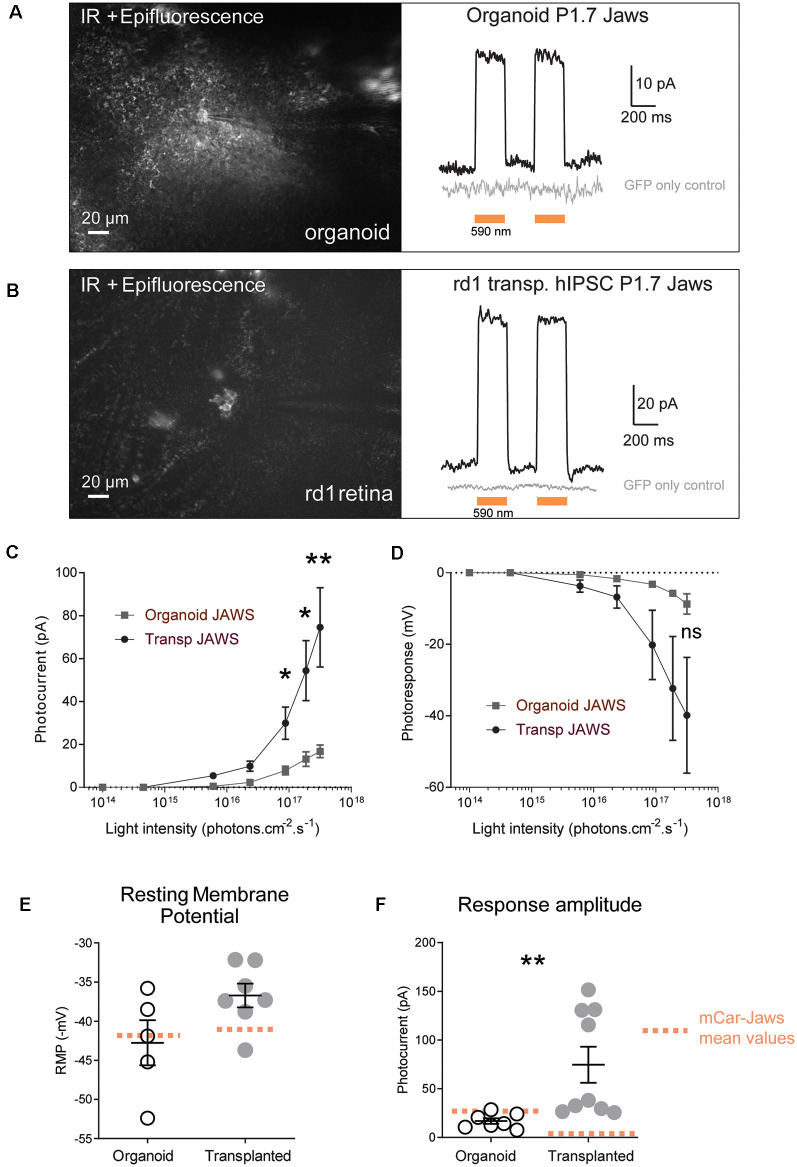
Jaws-driven light responses in engineered retinal organoids before and after transplantation. **(A)** *Left*. Infrared (IR)/epifluorescence image showing a transfected organoid with an electrode contacting a GFP+ cell. *Right*, photocurrent responses after stimulation with two consecutive flashes at a light intensity of 3.1 × 10^17^ photons cm^−2^ s^−1^, absence of response in GFP- cells is shown in gray. **(B)** *Left*. Infrared/epifluorescence-Infrared image of a rd1 transplanted retina depicting small clusters of fluorescent cells. *Right*, photocurrent responses after stimulation with two consecutive flashes at a light intensity of 3.1 × 10^17^ photons cm^−2^ s^−1^, absence of response in GFP-cells is shown in gray. **(C,D)** Light-intensity dependency of photoresponse amplitude. Photocurrent **(C)** and voltage hyperpolarization **(D)** of GFP+ cells in organoids and rd1 transplanted retinae as a function of light intensities ranging between 10^14^ and 3.1×10^17^ photons cm^−2^ s^−1^. **(E)** Resting membrane potential (RMP) of Jaws-expressing photoreceptors in the dark (at 0 current) for the recordings presented in panel **(D)**. Mean RMP values were −42, 76 ± 2, 88 mV in organoids and −36.72 ± 1.52 mV in transplanted cells. **(F)** Comparison of maximum photocurrent response amplitudes at the highest light stimulation intensity, for GFP+ cells in organoids (16.8 ± 2.92 pA) or after transplantation (74.57 ± 18.48 pA). Mean values obtained with our previous protocol (Garita-Hernandez et al., 2019) are depicted as doted orange lines. For all recordings the stimulation was performed at 590 nm if not stated otherwise. Values correspond to mean ± SEM. *p < 0.05, **p < 0.001, ns = not significant.

Next, we tested if Jaws-GFP positive cells can elicit light responses in response to illumination. Two-photon guided patch-clamp recordings revealed robust responses to orange light pulses (590 nm, 3.1 × 10^17^ photons.cm^−2^.s^−1^) in organoids (Figure 3A) and rd1 retinae after transplantation (Figure 3B). Jaws-elicited responses presented a typical photocurrent shape consisting of a fast onset (sometimes with a small transient) followed by a steady-state current. These light responses are based on the activity of the microbial opsin since organoids infected with control AAV-GFP did not elicit photocurrents upon stimulation. In the same way, age-matched transplanted mice with photoreceptors expressing GFP only showed no measurable light-evoked currents, which is consistent with the lack of outer segments in the transplanted cell population (gray traces in Figures 3A,B). Whole-cell patch-clamp recordings in GFP-Jaws cones exhibited robust light responses to orange light flashes, which increased continuously with increasing light intensity (Figures 3C,D). Of note, significant differences in photocurrents were found after transplantation compared to organoids starting at a stimulus intensity of 6 × 10^15^ photons.cm^−2^.s^−1^ (Figures 3A–F). Accordingly, hyperpolarization expressed as transmembrane voltage, increased steadily when increasing the light intensity up to 3.1 × 10^17^ photons.cm^−2^.s^−1^, with all the light intensities, used remaining below the safety limit for optical radiation in the human eye (European Parliament and European Council; International Commission on Non-Ionizing Radiation Protection). Importantly, the mean values of the resting membrane potential (RMP) in the dark (at 0 current) were −42.76 ± 2.88 mV in organoids and were even more depolarized at −36.72 ± 1.52 mV after transplantation which is higher than with our previous approach for transplanted cells (Figure 3E, orange dots; Garita-Hernandez et al., 2019). Those significantly depolarized RMP values are consistent with the results reported by Busskamp et al. who showed dormant cone cell bodies completely lacking outer segments were found to be surprisingly depolarized (Busskamp et al., 2010). Indeed, a depolarized state in the dark is mandatory in photoreceptors to activate calcium channels necessary for glutamate release. Additionally, we comparatively analyzed the amplitude of the photocurrents generated by PR1.7-Jaws in organoids and transplanted retinas vs. those observed by mCAR-Jaws used in our past studies (Garita-Hernandez et al., 2019). We observed significantly higher response amplitudes in the transplanted retinas than in those measured in organoids transformed with PR1.7-Jaws, significant response differences started at the intensity of 6 × 10^15^ photons.cm^−2^.s^−1^ as shown in Figure 3 and at the highest light intensity of 3.1 × 10^17^ photons.cm^−2^.s^−1^ we obtained 16.8 ± 2.91 pA in organoids (*n* = 7) and 74.57 ± 18.48 pA after transplantation (*n* = 9**, *p* = 0.0012, Mann–Whitney). More importantly, higher response amplitudes were recorded in retinae transplanted with PR1.7-Jaws when compared with mCAR-Jaws (Figure 3F) at the same intensity. By comparison, after transplantation with our precedent protocol, using mCAR promoter, we observed a mean response amplitude around 5 pA (Garita-Hernandez et al., 2019). Altogether, we show here that using a highly specific cone promoter we can increase Jaws expression levels in the cone membrane and observe an increase in light response amplitudes.

### Temporal Properties of Jaws-Induced Responses in hiPSC-Derived Cones After Transplantation

**Figure 4:**
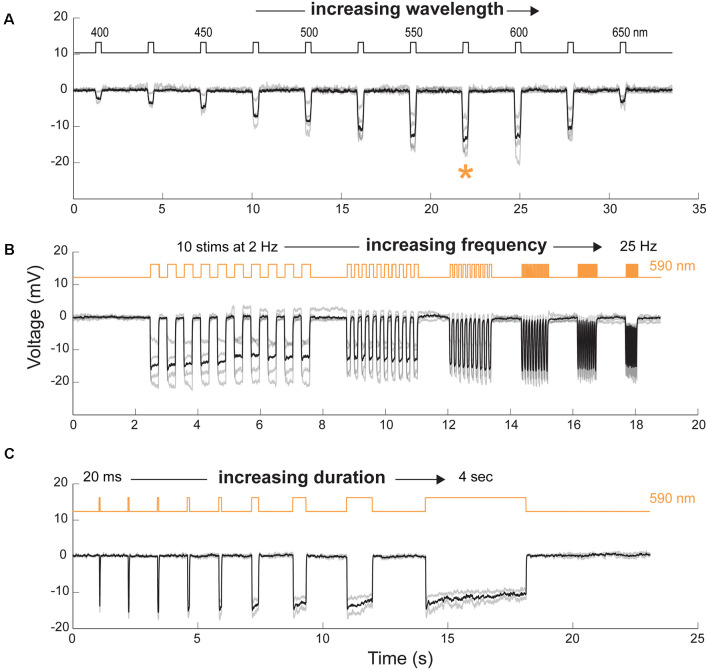
Spectral and temporal properties of transplanted cells. **(A)** Cone hyperpolarization induced by Jaws as a function of increasing wavelengths stimuli ranging from 400 to 650 nm. Maximal responses, when recorded at an intensity of 8 × 10^16^ photons cm^−2^ s^−1^, were obtained at a wavelength of 575 nm (orange asterisk). **(B)** Modulation of Jaws-induced voltage responses at increasing stimulation frequencies ranging from 2 to 25 Hz. **(C)** Modulation of Jaws-induced responses as a function of stimulus duration with stimuli ranging from 20 ms to 4 s in Jaws expressing cones at 8 × 10^16^ photons cm^2^ s^−1^. The timing and duration of stimulations are depicted with black lines in **(A)** or orange lines in **(B** and **C)** (590 nm stimuli). *n* = 4, 4 and 2 cells in **(A**, **B** and **C)**, respectively. Individual cell responses are represented in gray, mean response in black.

To further characterize the properties of Jaws-induced light responses, we used two-photon guided patch-clamp techniques to record hyperpolarization in single cone cells under current-clamp whole-cell configuration. The spectral sensitivity of Jaws-expressing cells was measured by stimulating the cells at different wavelengths. The action spectrum of Jaws, with maximal responses between 575 nm and 600 nm was confirmed (Figure 4A). To examine temporal properties of Jaws-cones, we first recorded light-responses using illumination pulses at increasing frequencies and observed that cells could follow up to 25 Hz stimuli with an excellent signal-to-noise ratio (Figure 4B). This is in line with our previous observations that optogenetically-engineered photoreceptors respond to light at a faster pace than natural photoreceptors. Then we recorded light responses as a function of stimuli of increasing durations, from 20 ms to 4 s. Cells’ responses were obtained at all durations, following the stimulus precisely, even at 4 s (Figure 4C). Because of the fast kinetics and the robustness of Jaws-cones responses, intermittent and complex stimuli can hence be applied and still elicit responses, thus also reducing drastically the total energy reaching the retina compared with a strategy that would use a single continuous light stimulation, which is important for clinical applications and light stimulation devices.

## Discussion

Genetic modification of cells within hiPSC-derived retinal organoids is essential to their utility as models of human organ development, as disease models, or for their therapeutic use as a source of cells for regenerative medicine. Genetic modifications to introduce small changes in the genome such as disease-causing mutations or the knock-out/integration of entire genes mostly use gene-editing tools at the single pluripotent stem cell stage, before selection and differentiation (Kruczek and Swaroop, 2020). However, the expression of exogenous genes to control cellular function after differentiation is needed for asking biological questions concerning disease mechanisms or using the cells for downstream therapeutic applications (Llonch et al., 2018). Moreover, the use of human retinal organoid models to test transgene expression has become a critical addition to the gene therapy pipeline bridging proof of concept work towards clinical applications by removing potential interspecies differences (Kalatzis et al., 2013; Cereso et al., 2014; Garita-Hernandez et al., 2018; Quinn et al., 2018). The specific features of transgene expression need to suit the ultimate purpose of the experiment both for gene therapy validation and transplantation. In our experiments, we sought to achieve a stable modification of the cone photoreceptor cell population within the human retinal organoids to render them light-sensitive using optogenetics. For our purpose, achieving detectable expression early in the cone cell population was necessary to isolate these cells before transplantation. Increasing the light sensitivity within the sorted cell population post-transplantation was a second desirable feature fit for our downstream application.

In advanced stages of retinal degeneration where photoreceptor cells are lost, a promising therapeutic strategy would be to transplant healthy and functional photoreceptors. For this type of therapy to be successful, transplanted photoreceptor cells need to respond to light and transmit the information to second-order neurons of the host retina. Moreover, chromophore replenishment through the support cells should be ensured. Despite several advances in the field of retinal differentiation (Meyer et al., 2009; Reichman et al., 2014, 2017; Zhong et al., 2014; Mellough et al., 2015; Garita-Hernandez et al., 2018; Capowski et al., 2019), functional light responses remain sparsely reported at the cellular level (Zhong et al., 2014; Hallam et al., 2018; Cowan et al., 2020). Moreover, transplantation studies using a photoreceptor cell suspension, in end-stage retinal degeneration, have not reported a fully functional outer segment. Importantly in the field, direct proof of transplanted cell functionality by direct patch-clamp photoreceptor cell recordings is rarely provided. In the case of retinal sheet transplantation, seldom has a developing outer segment been described with some MEA responses (Mandai et al., 2017; Iraha et al., 2018; McLelland et al., 2018), but RPE contact with donor cells has not been studied extensively at this stage. In our recent work, we proposed a new approach and demonstrated that the combination of stem cell-derived photoreceptors and optogenetics can overcome the above-mentioned issues. We have shown that optogenetically modified cone cells, expressing the microbial opsin Jaws under a mouse cone arrestin promoter can integrate into mouse retinas after the loss of endogenous photoreceptors and transmit the light signal to second and third-order neurons (Garita-Hernandez et al., 2019). This approach overcame the issues related to lack of outer segments and interaction with support cells, however, it required very high light levels for opsin activation and the expression levels were not compatible with cell sorting of the cone population before transplantation. In this work, we outweigh the limitations of our past work by a refined regulation of microbial opsin expression. Implementation of the strong PR1.7 cone promoter enhanced the expression of Jaws in 3D human retinal organoids and improved light responses especially after the cells matured in the subretinal space. Our work challenges the idea that photoreceptor precursors with integration capacity cannot be found at an earlier time point than other transplantation studies have shown, where the purification of a transplantable population has been based on cell surface markers that express later in differentiation using human pluripotent stem cells (Figure 1H; Gagliardi et al., 2018; Lakowski et al., 2018). It also highlights the importance of the promoter in driving transgene expression in retinal organoids both in terms of kinetics and expression levels.

## Data Availability Statement

Requests to access the datasets should be directed to marcela.garita@inserm.fr.

## Ethics Statement

The animal study was reviewed and approved by local animal experimentation ethics committee (Le Comité d’Ethique pour l’Expérimentation Animale Charles Darwin) and National Institutes of Health guide for the care and use of laboratory animals as well as the Directive 2010/63/EU of the European Parliament.

## Author Contributions

MG-H optimized the generation and AAV mediated transduction of hiPSC-derived retinal organoids, performed culture, imaging, histology, designed experiments, and wrote the manuscript. AC performed patch-clamp recordings and 2-photon imaging and wrote the manuscript. LG generated hiPSC-derived retinal organoids, performed AAV infections, FACS, histology, imaging, subretinal injections, and fundus imaging. FR generated hiPSC derived retinal organoids, performed AAV infections, histology, and fundus imaging. HK performed subretinal injections. LR helped to optimize the FACS cell sorting. LT assisted in cell culture and AAV infections. LG, FR, and LT helped to draft the manuscript. SP and J-AS provided scientific input. OG provided hiPSCs, provided scientific input, and feedback on the manuscript. JD provided scientific input. DD designed the experiments, provided scientific input, and wrote the manuscript. All authors contributed to the article and approved the submitted version.

## Conflict of Interest

MG-H, AC, OG, DD, JD, and J-AS are inventors on a patent on the use of hiPSC to treat retinal degeneration (WO2018055131). DD is an inventor on a patent of adeno-associated virus virions with variant capsid and methods of use thereof with royalties paid to Avalanche Biotech (WO2012145601 A2). DD and HK are inventors on pending patent applications on noninvasive methods to target cone photoreceptors (EP17306429.6 and EP17306430.4) licensed to Gamut Tx. DD, HK and OG are founders of Gamut Tx. OG and J-AS are inventors on a patent on hiPSC retinal differentiation (WO2014174492 A1). J-AS is a founder and consultant for Pixium Vision and GenSight Biologics and is a consultant for Sanofi-Fovea, Genesignal, and Vision Medicines. The remaining authors declare that the research was conducted in the absence of any commercial or financial relationships that could be construed as a potential conflict of interest.
