# Supplementary figures and images for: Control of Microbial Opsin Expression in Stem Cell Derived Cones for Improved Outcomes in Cell Therapy
Source: Front Cell Neurosci. 2021 Mar 18;15:648210. doi: 10.3389/fncel.2021.648210 (PMC8012682; doi:10.3389/fncel.2021.648210)

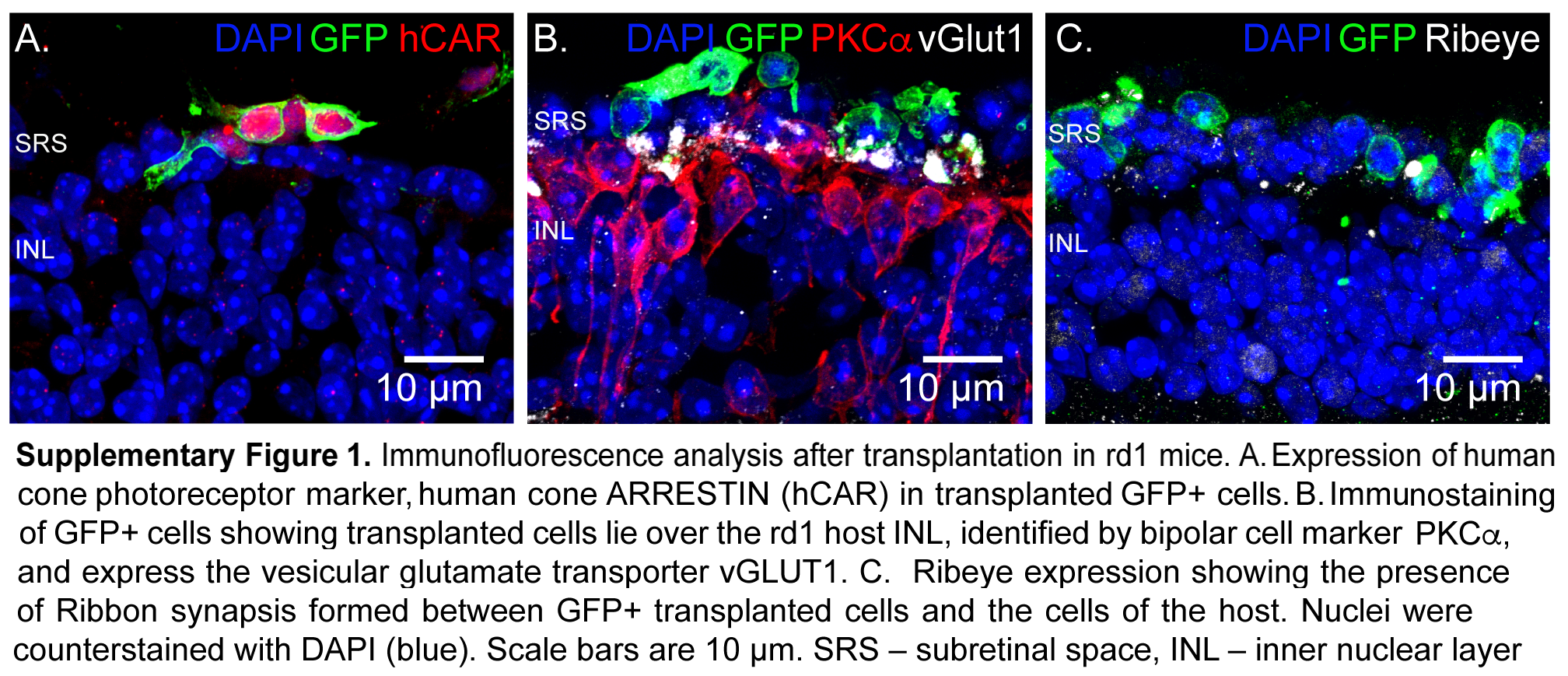

Supplement: Supplementary file 1 [file Image_1.TIF]
